# Resist-free antireflective nanostructured film fabricated by thermal-NIL

**DOI:** 10.1186/s40580-014-0019-1

**Published:** 2014-05-20

**Authors:** Young Hun Kang, Jae Hyung Han, Song Yun Cho, Choon-Gi Choi

**Affiliations:** 1 Creative Research Center for Graphene Electronics, Electronics and Telecommunications Research Institute (ETRI), Daejeon, 305-700 Korea; 2 Division of Advanced Materials, Korea Research Institute of Chemical Technology, Daejeon, 305-600 Korea

**Keywords:** Antireflective films, Moth-eye, Nanostructures, Anodic aluminum oxide template, Thermal nano-imprint lithography, Oxygen reactive ion etching

## Abstract

Resist-free antireflective (AR) nanostructured films are directly fabricated on polycarbonate (PC) film using thermal-nanoimprint lithography (T-NIL) and the moth-eye shape of AR nanostructure is elaborately optimized with different oxygen reactive ion etching conditions. Anodic aluminum oxide (AAO) templates are directly used as master molds of T-NIL for preparation of AR nanostructures on PC film without an additional T-NIL resist. AR nanostructures are well arranged with a period of about 200 nm and diameter of about 150 nm, which corresponds to those of the AAO template mold. The moth-eye AR nanostructures exhibit the average reflectance of 2% in wavelength range from 400 to 800 nm. From the results, highly enhanced AR properties with simple direct imprinting on PC film demonstrate the potential for panel application in the field of flat display, touch screen, and solar cells.

## Background

Recently, an antireflection (AR) property has been a great interest due to the demands for the enhanced performance in electronic devices such as solar cells and displays [1]. Especially, needs for AR property of plastic substrate have been increasing for the rapid growth of flexible electronic fields. Many research groups have already investigated various AR techniques such as single or multi-layer coating, surface texturization, and fabricating the periodic nanostructures [2]. AR nanostructure, so-called moth-eye structure, can efficiently reduce the light reflectance attributed to the gradual change in refractive index between the air and substrate by altering the volume fraction of structures, which in turn reduce Fresnel reflection [3]. There are many techniques available for fabricating AR nanostructure, such as electron beam lithography [4], thermal imprinting [5], hybrid nano-patterning lithography [6], dip pen nanolithography [7] and so on. However, these techniques still require high cost, complex process, and sophisticated equipments. In order to overcome these drawbacks, we employ the nanoimprint lithography (NIL) which is one of the most potential nano-scale patterning techniques with advantages of rapidness, low cost, and reproducibility. In NIL process, molds or stamps are normally produced using various materials, such as silicon, glass, and metal. However, it is difficult to prepare the periodically uniform nano-sized patterns on large area using molds from these materials. Therefore, an anodic aluminum oxide (AAO) template can be good candidate for mold material due to the advantages of the facile large scale synthesis via electrochemical method and periodical nano-sized patterns with high aspect ratio [8].

In this study, we report a facile method for the preparation of AR nanostructure on PC film using AAO template as a master mold through the T-NIL process. Furthermore, the shape of AR nanostructures is elaborately optimized with different reactive ion etching (RIE) conditions and the optical properties of the fabricated AR films are investigated.

## 2 Methods

### Experiment

**Figure 1:**
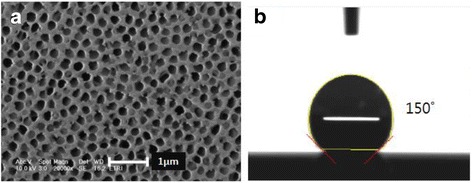
**AAO template: (a) SEM image of the surface of AAO template and (b) contact angle of the AAO template after anti-sticking treatment.**

To fabricate AR nanostructures on PC film, a commercial AAO template (Whatman Inc.) fabricated by a multi-step anodising process was used as template mold. As shown in Figure 1(a), the pore diameter of AAO template was approximately 200 nm and the size of AAO template was 2 × 2 in^2^.

Prior to T-NIL process, AAO templates were coated with the anti-adhesion layer to decrease the surface free energy of AAO for the easy separation of the AAO templates from PC film. A liquid phase deposition of *1H, 1H, 2H, 2H*-perfluorooctyl-trichlorosilane was used to form the self-assembled monolayer (SAM) based anti-adhesion layer. The formation of hydrophobic anti-adhesion layer on the surface of the AAO templates was confirmed by contact angle measurement which was determined to be 150° as shown in Figure 1(b).

**Scheme 1:**
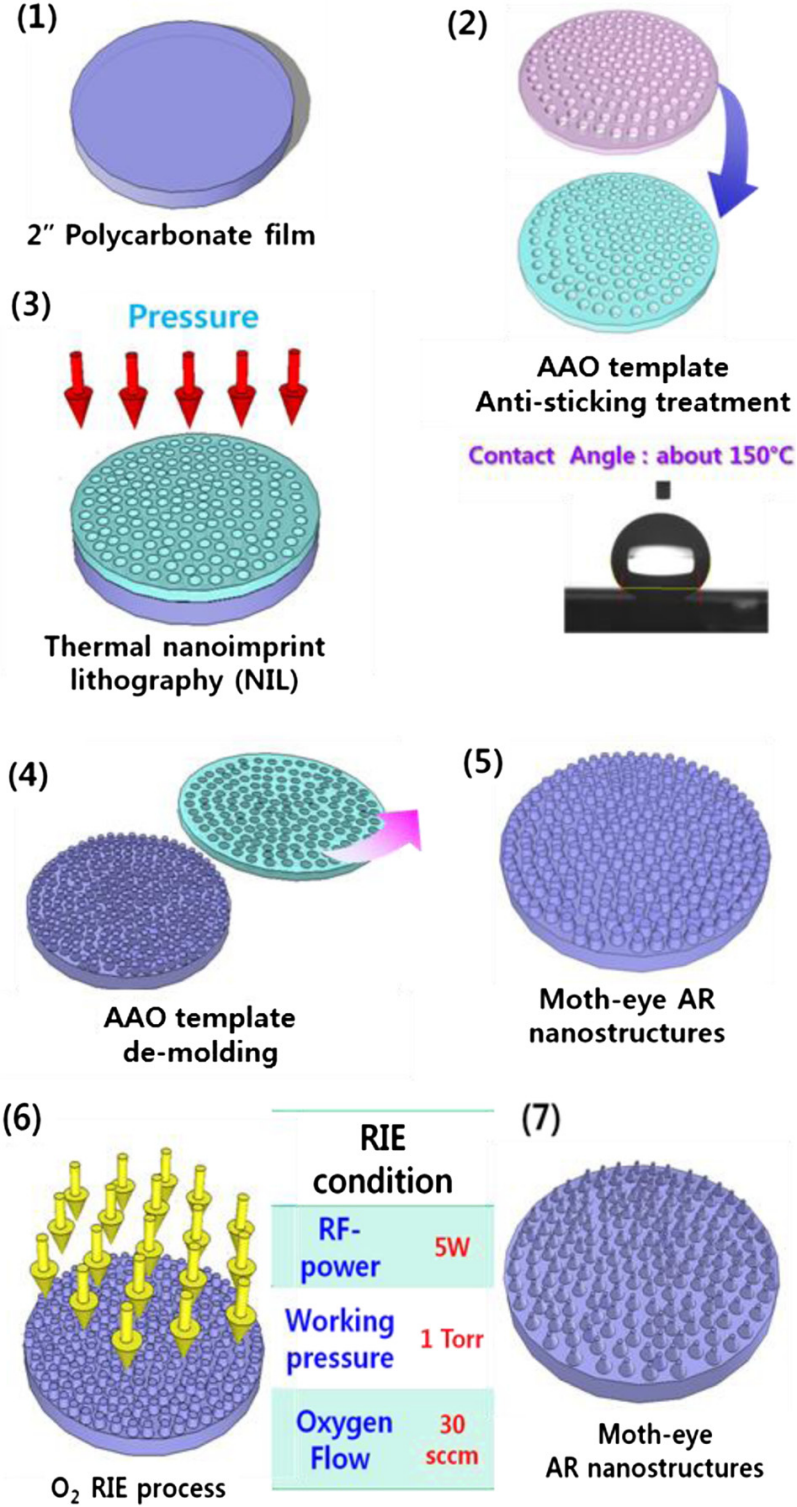
**Schematic diagram of T-NIL and RIE process for fabrication of AR nanostructured films.**

T-NIL process was carried out on PC film with Nano-Imprinter (Obducat, Sweden) using AAO template mold under the various temperatures (140, 150, 160, and 170°C) and pressure (8, 10, and 12 Bar) for 20 min. A home-made PC film was used and the thickness of PC film is about 1 mm. After removing the AAO template mold, oxygen RIE process was optimized for the proper formation of AR nanostructure on PC film with the various parameters, which were oxygen flow of 30 sccm under the power of 5 W at different etching time (60, 120, and 180 s). Surface morphology of the imprinted AR nanostructures on PC film was investigated by field emission scanning electron microscope (FE-SEM) and the optical reflectance of the imprinted PC film was measured by UV-visible spectrophotometer (Hitachi U-3501). The schematic procedure for the preparation of the AR nanostructure on PC film is illustrated in Scheme 1.

## Results and discussion

**Figure 2:**
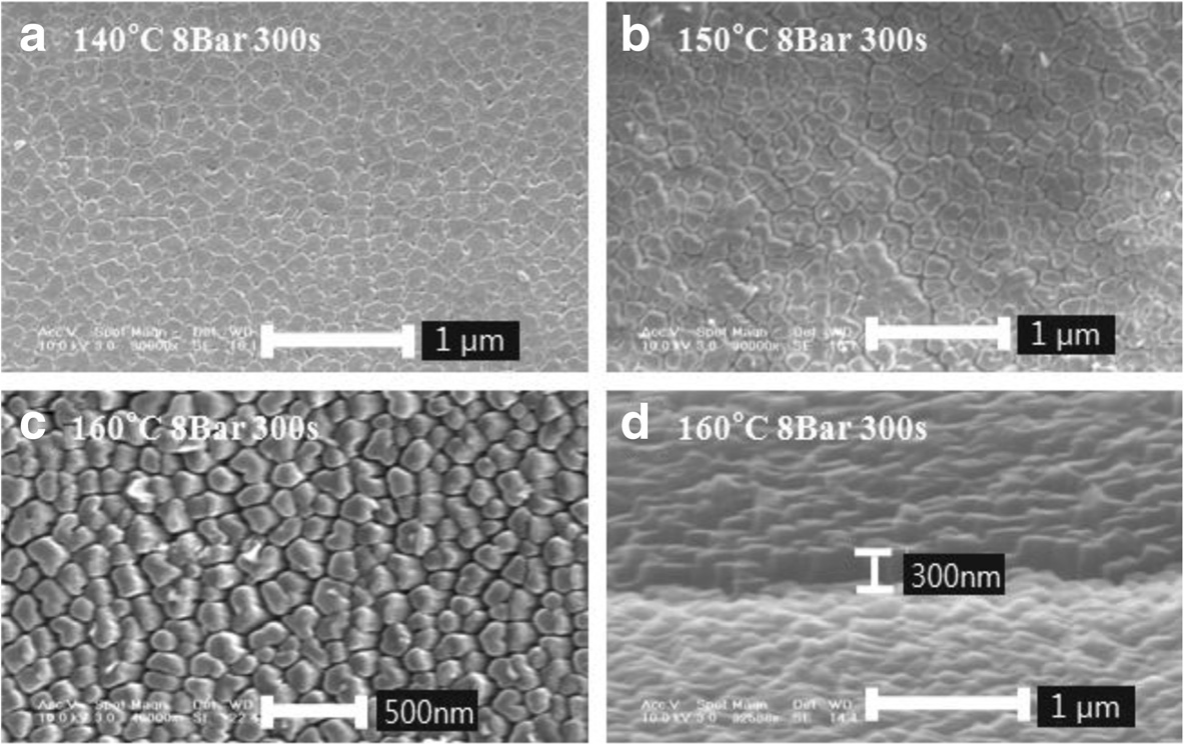
**FE-SEM images of nanoimprinted AR nanostructures with various process temperatures: (a) 140°C, (b) 150°C, (c) 160°C, and (d) the tilted view of the imprinted AR nanostructures at temperature of 160°C.**

Figure 2 shows the FE-SEM images of the nanoimprinted AR nanostructure on PC film by T-NIL. Although the pore shape of AAO templates was clearly observed on the surface of PC films, AR nanostructure is roughly formed at a pressure of 8 bar, as shown in Figure 2(a) and (b). It is attributed that the periodic nano-sized pores of AAO templates were difficult to be filled with PC at too low process temperature. Whereas, AR nanostructure fabricated at 160°C shows a period and height of 200 and 300 nm, respectively with a diameter about 150 nm, as shown in Figure 2(c) and (d). These pattern properties on PC well correspond with those of AAO template. This result means that nanoimprinting process for AR nanostructure on PC is highly dependent on processing temperature and AR nanostructures can be completely replicated on PC film at 160°C. However, there exist the problem of separation between AAO template and PC film for high temperature process over 160°C due to the intense adhesion between inner walls of AAO nano-pores and the melted PC film, which results in the structural damage of AR nanostructures.

**Figure 3:**
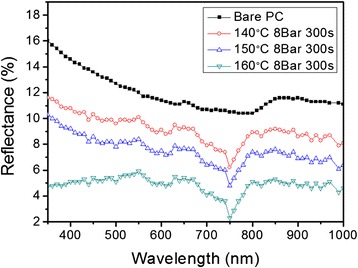
**Reflectance of the AR nanostructures nanoimprinted on the PC film in the wavelength range of 400 nm - 1,000 nm measured by a UV-visible spectrophotometer.**

To evaluate the AR properties of nanoimprinted PC films, the reflectance was measured by UV-visible spectroscopy in the wavelength range from 400 nm to 1,000 nm, as shown in the Figure 3. It is clearly observed that the AR nanostructures prepared at 160°C exhibits the average reflectance of about 5%. In comparison with that of the bare PC film, the average reflectance was considerably reduced from 13% to 5% in the visible light range. At 140 and 150°C, the average reflectance of the AR nanostructures is about 7% and 9%, respectively because periodic AR nanostructures were not clearly formed and separated.

**Figure 4:**
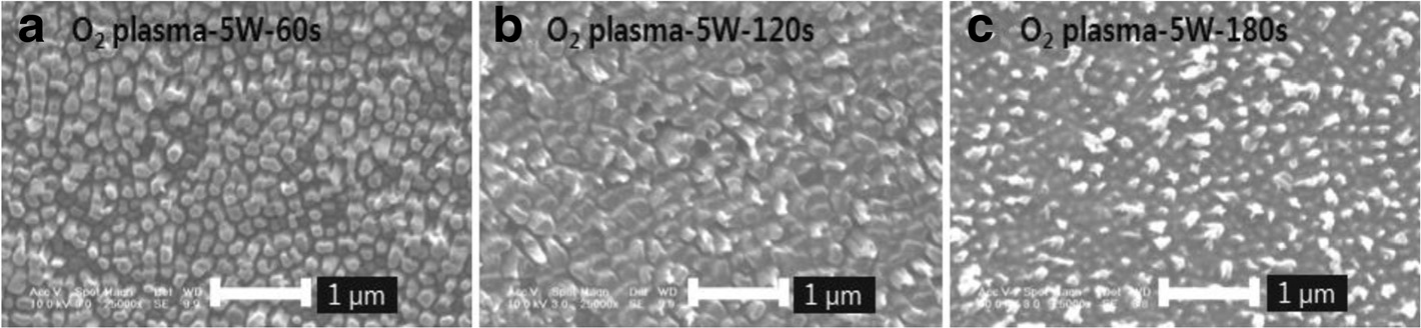
**FE-SEM images of the AR nanostructures after O**_**2**_ **RIE process with different etching time.**

In order to optimize the shape of AR nanostructures and reduce the reflectance, AR nanostructures fabricated at 160°C by T-NIL was etched by the oxygen RIE process with the various etching time. It is observed that oxygen RIE process has great influence on the surface morphology of AR nanostructures, as shown in Figure 4. The AR nanostructures which were formed by RIE process for 60 s obviously represents the pillar shape, as shown in Figure 4(a). However, with the increase of the etching time over 60 s, AR nanostructures are collapsed due to the prolonged ion bombardment during the RIE process. At 180 s of the etching time, as shown in Figure 4(c), the period of AR nanostructures is increased and the pillar shape is changed with the decrease of height and width.

**Figure 5:**
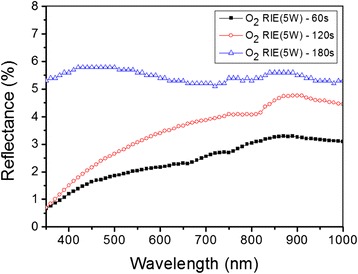
**Reflectance of AR nanostructures on the PC film after oxygen RIE process with various etching time.**

Figure 5 shows the reflectance of AR nanostructures after oxygen RIE process. The average reflectance of AR nanostructures after oxygen RIE process with 60 s etching time exhibits about 2% in the visible light range from 400 nm to 800 nm. In compared with that of the AR nanostructures for the etching time of 180 s, the average reflectance is also decreased from 5% to 2%. Therefore, the average reflectance of AR nanostructures with additional 60 s RIE etching time is significantly decreased from 5% to 2% in the 400–800 nm visible range in comparison with that of the nanoimprinted AR nanostructures at 160°C.

**Figure 6:**
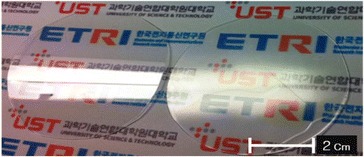
**Digital images of the bare PC film (left) and the AR nanostructured PC film (right).**

The reflectance difference between the bare PC film and PC film with oxygen RIE processed AR nanostructures is demonstrated visually in Figure 6. The film on the left-hand side is the bare PC film and the film on the right-hand side is the AR nanostructured PC film. The bare PC film has strong surface reflection because of no AR on the surface. On the contrary, no noticeable surface reflection can be observed with the AR nanostructured PC film. Therefore, it is observed that PC film with oxygen RIE processed AR nanostructures can obviously transmit the text of printed page due to the significant reduction of light reflectance. These results highlight the potential of fabricating a high performance AR film by forming moth-eye nanostructures on polymer films.

## Conclusions

We successfully demonstrate that the resist-free AR nanostructures are directly fabricated on PC film using T-NIL and the shape of AR nanostructures is elaborately optimized with different oxygen RIE conditions. AAO templates are directly used as master molds of T-NIL for preparation of AR nanostructures on PC film without an additional T-NIL resist. AR nanostructures are well arranged with a period of about 200 nm and diameter of about 150 nm, which corresponds to those of the AAO template mold. The average reflectance of the PC film with AR nanostructures was obtained to be about 2% in the broadband wavelength range of 400–800 nm. Comparing to bare PC film and the fabricated AR film, the average reflectance of AR film is reduced to approximately 11% from 13% to 2% in the visible light region. As a result, moth-eye AR nanostructured PC film using T-NIL and oxygen RIE can efficiently reduce the light reflectance at the visible light region. We suggest that the prepared AR film is useful for panel application in the field of flat display, touch screen, and solar cells.
